# Melatonin as an Anti-Aging Therapy for Age-Related Cardiovascular and Neurodegenerative Diseases

**DOI:** 10.3389/fnagi.2022.888292

**Published:** 2022-06-03

**Authors:** Virna Margarita Martín Giménez, Natalia de las Heras, Vicente Lahera, Jesús A. F. Tresguerres, Russel J. Reiter, Walter Manucha

**Affiliations:** ^1^Instituto de Investigaciones en Ciencias Químicas, Facultad de Ciencias Químicas y Tecnológicas, Universidad Católica de Cuyo, San Juan, Argentina; ^2^Departamento de Fisiología, Facultad de Medicina, Universidad Complutense, Madrid, Spain; ^3^Department of Cell Systems and Anatomy, UT Health San Antonio Long School of Medicine, San Antonio, TX, United States; ^4^Área de Farmacología, Departamento de Patología, Facultad de Ciencias Médicas, Universidad Nacional de Cuyo, Mendoza, Argentina; ^5^Instituto de Medicina y Biología Experimental de Cuyo (IMBECU), Consejo Nacional de Investigaciones Científicas y Tecnológicas (CONICET), Mendoza, Argentina

**Keywords:** aging, mitochondria, oxidative stress, inflammation, melatonin, neurodegenerative disease, cardiovascular diseases

## Abstract

The concept of “aging” is defined as the set of gradual and progressive changes in an organism that leads to an increased risk of weakness, disease, and death. This process may occur at the cellular and organ level, as well as in the entire organism of any living being. During aging, there is a decrease in biological functions and in the ability to adapt to metabolic stress. General effects of aging include mitochondrial, cellular, and organic dysfunction, immune impairment or inflammaging, oxidative stress, cognitive and cardiovascular alterations, among others. Therefore, one of the main harmful consequences of aging is the development and progression of multiple diseases related to these processes, especially at the cardiovascular and central nervous system levels. Both cardiovascular and neurodegenerative pathologies are highly disabling and, in many cases, lethal. In this context, melatonin, an endogenous compound naturally synthesized not only by the pineal gland but also by many cell types, may have a key role in the modulation of multiple mechanisms associated with aging. Additionally, this indoleamine is also a therapeutic agent, which may be administered exogenously with a high degree of safety. For this reason, melatonin could become an attractive and low-cost alternative for slowing the processes of aging and its associated diseases, including cardiovascular and neurodegenerative disorders.

## Introduction

Melatonin is an endogenous indoleamine that is synthesized not only in the pineal gland (endocrine gland located in the posterior area of the cranial fossa in the brain in charge of producing melatonin and serotonin-derived hormones crucial in the modulation of sleep patterns in both circadian and seasonal cycles) but also perhaps in each individual cell, specifically at the mitochondrial level (Yanar et al., 2019). Independently of its production site, melatonin levels usually vary depending, among other factors, on the age of the organism in which is synthesized. Thus, during aging, there is a drastic mitochondrial loss of thioretinacoozonide, a complex that binds the phosphate groups of adenosine triphosphate and oxygen in the oxidative phosphorylation process. As a result, adenosyl methionine (methyl donor obtained from thioretinacoozonide and ATP, which is involved in melatonin synthesis from N-acetyl serotonin) levels are reduced and, consequently, melatonin production also diminishes. These changes lead to altered oxidative phosphorylation, as well as increased oxidative stress, calcium influx, inflammation, apoptosis, and mitochondrial dysfunction, being all of them physiopathological processes commonly observed in aging and its associated diseases (McCully, 2018). Particularly, mitochondrial dysfunction is characterized by reduced respiratory complex activity, increased free radical generation, nitric oxide (NO) production, and mitochondrial synthase activity, as well as by impaired electron transport system and/or mitochondrial permeability. To highlight, a 10-fold decrease in pineal melatonin production in octogenarians compared to teenagers was observed, which results in a significant attenuation of the antioxidant, anti-inflammatory, and mitochondrial optimizing effects of melatonin (Melhuish Beaupre et al., 2021). This has important consequences for the immune changes common over the course of aging, including immune senescence and inflammaging (inflammation associated with aging), which may be mediated by the effects of night-time pineal melatonin in resetting and resynchronizing immune cell mitochondrial function (Anderson, 2019; Reiter et al., 2019). In this context, exogenous melatonin may act as a powerful antioxidant and, consequently, anti-aging agent due to its ability to re-establish the mitochondrial membrane permeability and to stimulate antioxidant enzymes such as glutathione peroxidase, superoxide dismutase, glutathione reductase, and catalase, among others. Melatonin is also an inhibitor of lipoxygenase enzyme and may promote resistance against oxidative damage by repairing the microsomal membranes (Sharafati-Chaleshtori et al., 2017). Additionally, the exogenous administration of melatonin has been shown to increase the expression of suppressors of cytokine signaling, thus enhancing the immune response and contributing to the anti-aging effects of this compound (Tresguerres et al., 2012; Vinod and Jagota, 2017). Consequently, melatonin has even been proposed as a molecule potentially capable of extending lifespan by allowing healthy aging (Karadas et al., 2019).

In addition to the described events at the cellular level, aging also promotes the dysfunction of the pineal gland, which is, as mentioned, an important source of melatonin, thus affecting several physiological processes mainly related to circadian rhythms. It has been suggested that this phenomenon is a consequence of lipid peroxidation on Elongation Factor 2 (a key component of protein synthesis machinery) in the pineal gland. In this regard, it has been observed that the administration of exogenous melatonin also may prevent pineal dysfunction associated with aging, thus avoiding or delaying several alterations that accompany this process (Muñoz et al., 2017).

Hence, a vicious circle between melatonin production levels and aging seems to exist, since melatonin synthesis decreases with the age and, at the same time, the aging is worsened as a consequence of the melatonin deficiency (Hardeland, 2019). In fact, the relationship between melatonin and aging is so strong that both extrapineal and pineal melatonin are currently considered useful markers of the aging rate of an organism. Therefore, melatonin levels may be a predictor or an indicator of numerous diseases associated with aging (Konovalov et al., 2017; Melhuish Beaupre et al., 2021). For example, reduced levels of melatonin in buccal cells, blood plasma, and other human samples have been correlated with patient age, as well as with the development of Alzheimer’s disease and the severity of the menopausal syndrome, among other aging-derived pathologies or conditions (Zuev et al., 2017).

In this context, melatonergic signaling attenuated during aging has been associated with the development or acceleration of neuropsychiatric disorders and alteration of different neuronal processes such as cognition, memory, neurogenesis, and neurorestorative mechanisms (Bahna and Niles, 2018). Likewise, a deficiency of endogenous melatonin has also been observed with the pathogenesis of cardiovascular disorders, including myocardial infarction, cardiac hypertrophy, vascular dysfunction, lethal cardiac arrhythmias, vascular calcification, atherosclerosis, ischemia/reperfusion injury, stroke, among other age-derived pathologies (Mocayar Marón et al., 2020; Ozkalayci et al., 2021).

Given what is known about aging, it has been suggested that integrated therapeutic alternatives should be explored to slow the aging rate in order to prevent or delay more efficiently, age-related diseases instead of treating them individually, which is the most common pharmacological approach currently used (Vaiserman et al., 2021). As a result, exogenous melatonin has been proposed as a multitasking molecule capable of retarding several processes related to aging such as circadian rhythm dysregulation, metabolic alterations, inflammation, oxidative stress, and mitochondrial dysfunction. The anti-inflammatory and anti-oxidative effects of melatonin, especially at the mitochondrial level, may exert protective actions against inflammaging processes. Thus, exogenously administered melatonin may prevent and mitigate multiple cardiovascular diseases, as well as neurodegenerative pathologies such as Parkinson’s and Alzheimer’s diseases, whose development and advance are preceded and influenced by inflammaging events (Paredes et al., 2014; Cardinali and Hardeland, 2017; Korábečný, 2018; Majidinia et al., 2018; Zhong and Liu, 2018; García et al., 2020; Vaiserman et al., 2021).

For all these reasons, in this review, we analyze the close relationship that exists between melatonin levels and the aging process, as well as summarize the most recent findings on the therapeutic use of exogenous melatonin in the prevention and treatment of cardiovascular and neurodegenerative diseases as relevant examples of age-related pathologies.

## Etiology and Pathophysiology of Aging

Aging is associated with a progressive decline in numerous physiological processes, leading to an increased risk of health complications and disease in all systems and organs (Bulterijs et al., 2015). The increased longevity that is being experienced by the world population in recent years has meant going from a life expectancy at birth of 33 years, a century ago, to more than 80 years today (World Health Organization, WHO; Stambler et al., 2018). This increase in life expectancy is accompanied by the manifestation of ailments, diseases, and/or alterations that at younger ages did not occur or appeared very sporadically (López-Otín et al., 2013). Many of the diseases related to aging are associated with organic wasting caused by increased longevity, and general deterioration of the functions of the organism that leads to a lower ability to react to changes and preserve homeostasis adaptively (López-Otín et al., 2013). The rate at which homeostatic systems decline is directly linked to the degree of chronic oxidative and inflammatory processes. Many theories were proposed to explain how the process of aging occurs. Among them, the free radical theory of aging proposed by Harman (1956) and further developed by several authors (Harman, 1956, 1972; Miquel, 1998; Barja, 2013) is probably the most widely accepted one. This theory postulates that the production of intracellular reactive oxygen species (ROS) is the major determinant of aging and lifespan (Harman, 1956). At present, oxidative stress because of elevated ROS production and impaired antioxidant defense mechanisms, are still considered to be key contributors to the aging process.

There are several sources of cellular ROS including the endoplasmic reticulum, nucleus, peroxisomes, and the Golgi apparatus. These ROS sometimes act as second messengers and coordinate several molecular pathways within the cells. The mitochondrial free radical theory of aging proposed that the mitochondrial rate of ROS (mitROS) production is the most relevant feature of the aging process (Harman, 1972; De la Fuente and Miquel, 2009; Barja, 2013, 2019). One reason for the elevated ROS is the damage to mitochondrial DNA caused by mtROS since this DNA is highly vulnerable to oxidative damage because it lacks histones (Singh et al., 2015). As a consequence, the damage to mtDNA alters mitochondrial homeostasis and function of these organelles, thereby influencing cellular function. In addition, altered mtDNA fragments can insert into the nuclear DNA thereby amplifying cell damage (Puertas and González-Sánchez, 2020). Mammalian target of rapamycin (mTOR), along with other proteins and intracellular signaling pathways such as PPAR-gamma coactivator 1-alpha (PGC-1α), sirtuins, nuclear respiratory factor 1 (NRF1), AMPK, mitochondrial transcription factor A (TFAM), and PTEN-induced kinase 1(PINK)/E3 ubiquitin ligase (PARKIN) are involved in the mitochondrial quality control, which in turn depends on the coordination between mitochondrial biogenesis, mitochondrial dynamism, and mitophagy (Vives-Bauza et al., 2010; Lahera et al., 2017; Gureev et al., 2019).

Numerous data suggest a close link between oxidation and inflammation, since excessive oxidative stress induces an inflammatory response, and ROS are considered to be effectors of inflammation (De la Fuente and Miquel, 2009). The chronic inflammation is characterized by the infiltration of mononuclear immune cells into different tissues where these cells produce an excess of ROS and proinflammatory mediators. The persistent and active oxidant response by immune cells leads to cell damage due to the overproduction of ROS, which also recruits other inflammatory cells to the inflamed site causing an additional production of pro-inflammatory and oxidizing agents that amplify cell damage (Federico et al., 2007). In addition, aging it is accompanied by a progressive dysregulation of the immune system, mainly due to alterations in the cellular/adaptive immune response (Douziech et al., 2002). This imbalance commonly leads to the establishment of a low-grade inflammatory state in the elderly. The cause of this inflammatory state is multifactorial, although the origin may be chronic antigenic stimulation by bacteria or viruses or endogenous cellular factors (Fulop et al., 2017).

There are several pathways that mediate the connection between the inflammatory process and oxidation. Based on the idea described above related to the production of mitROS and altered mtDNA fragments, these fragments are known to act as damage-associated molecular patterns (DAMPs) that can bind to pattern recognition receptors (PRR) and, *via* activation of nuclear transcription factor kappa B (NF-κB), stimulate the expression of pro-inflammatory cytokines, which promote aging (Patel, 2018). In addition, mitROS can activate the NOD-like receptor family pyrin domain-containing 3 (NLRP3) inflammasome, leading to the processing and secretion of proinflammatory cytokines such as interleukin-1 and 18 (Meyers and Zhu, 2020).

The detrimental role of chronic inflammation during aging is further supported by clinical data (Libby, 2002; Yin et al., 2016; Klohs, 2019; Laderoute, 2020). A low level of chronic inflammation is associated with many diseases such as atherosclerosis, cardiovascular and neurodegenerative diseases, and diabetes. In fact, upregulation of the inflammatory response and the ensuing low-grade chronic systemic proinflammatory state underlies perhaps all age-associated diseases. Aging is accompanied by immune, hormonal, and adipose changes that lead to a chronic systemic inflammatory state, and may be considered the pillar of the pathophysiological process of frailty (Soysal et al., 2016). These changes likely influence the development of physical frailty, cognitive decline as well as negative cardiovascular events. As mentioned previously, the increased levels of chronic inflammation in these instances are termed inflammaging (Franceschi and Campisi, 2014), a highly significant risk factor for morbidity/mortality in the elderly, given that age-related diseases share an inflammatory pathogenesis. To date, various sources of inflammaging have been described, including macromolecules and damaged cells that accumulate with age due to increased production and/or inadequate elimination; cellular senescence in response to damage and stress; age-related alterations in the immune system, also known as immunosenescence; alterations in the complement pathway that are the cause of many degenerative diseases; and even harmful products produced by the oral or gut microbiome (Royce et al., 2019; Laderoute, 2020). This last aspect results of a great importance, since the gut microbiome is also intimately linked to many aging-associated conditions, including neurodegenerative (Cheng et al., 2022) and cardiovascular diseases (Anderson and Mazzoccoli, 2019; Akyildiz et al., 2022). Many of the beneficial effects of the gut microbiome are mediated *via* the production of the short-chain fatty acid, butyrate, which optimizes mitochondrial function, with effects that seem to require the upregulation of the melatonergic pathway (Anderson and Maes, 2020). Butyrate also increases intestinal melatonin synthesis (Song et al., 2021), indicating that optimized gut microbiome function may be intimately linked to the regulation of the melatonergic pathway. Such data highlights the important role that melatonin has in the regulation of wider aspects of aging-linked pathophysiology.

In addition, recent data have implicated the aryl hydrocarbon receptor (AhR) in many aging-linked conditions (Salminen, 2022). The AhR is activated following the production of pro-inflammatory cytokines that induce indoleamine 2,3-dioxygenase (IDO) leading to the conversion of tryptophan to kynurenine, with the latter activating the AhR (Mezrich et al., 2010). The consequent depletion of tryptophan will suppress its availability for the synthesis of serotonin, N-acetylserotonin, and melatonin, thereby allowing inflammation-driven AhR activation to suppress the endogenous melatonergic pathway. This is relevant in a number of aging-linked medical conditions, including neurodegenerative and cardiovascular diseases, as well as most cancers (Anderson and Reiter, 2019). Such data highlights the role of melatonergic pathway suppression in the pathoetiology of aging-linked medical conditions.

By the year 2030, it is estimated that 20% of the population will be 65 years of age or older. In this age group, cardiovascular and neurodegenerative diseases will cause more than 50% of all deaths and will be among its leading causes. In addition, the cost of treatment for these diseases will triple (Fleg et al., 2011; Pal et al., 2015). Thus, improving healthy aging in this large cohort would hot only improve the quality of life of the elderly but would also ease the financial burden of the associated treatments.

## Cardiovascular Pathologies Associated with Aging

The proper functioning of the cardiovascular system is crucial for the health of every tissue and organ since it delivers oxygen and nutrients while removing catabolites from every cell. The Framingham Heart Study and the Baltimore Longitudinal Study on Aging (BLSA) showed that in healthy individuals without concomitant cardiovascular diseases, aging results in an increase in the prevalence of left ventricular hypertrophy (LVH) and fibrosis, a decline in the diastolic function, and an increase in the prevalence of cardiac arrhythmias (Lakatta and Levy, 2003b). The excitability of the heart is also affected by aging. Heart rate is hampered by the loss of cells in the sinoatrial node, and the propagation of electric impulse is slowed due to structural changes in the heart (fibrosis and hypertrophy; Chiao and Rabinovitch, 2015). There is also an age-related increase in arterial intima/media thickening which is associated with vessel stiffness (Lakatta and Levy, 2003a). Deterioration of both the vascular system and the heart are mutually dependent. Arterial stiffness leads to increased peripheral resistance which develops compensatory mechanisms by the myocardium such as LVH and fibroblast proliferation. These cardiovascular alterations seem to be independent of traditional risk factors such as smoking, hypertension, increased circulating lipoprotein levels, diabetes, etc., and may be considered as part of intrinsic cardiovascular aging; they are, in part, age-related processes (North and Sinclair, 2012).

Gender differences in the incidence of aging-related cardiac diseases have been demonstrated. Men develop ischemic heart disease earlier than women. However, hypertension, acute coronary syndrome, and heart failure with preserved ejection fraction (HFpEF) are more common in aging women (Regitz-Zagrosek et al., 2016). Although the molecular mechanisms underlying these differences are not clear, data from experimental models suggests that estrogen deficiency results in the activation of several regulatory mechanisms such as the renin-angiotensin-aldosterone axis, which lead to the development of cardiac hypertrophy and fibrosis (Benjamin et al., 2019).

### Aging-Related Heart Alterations

**Figure 1:**
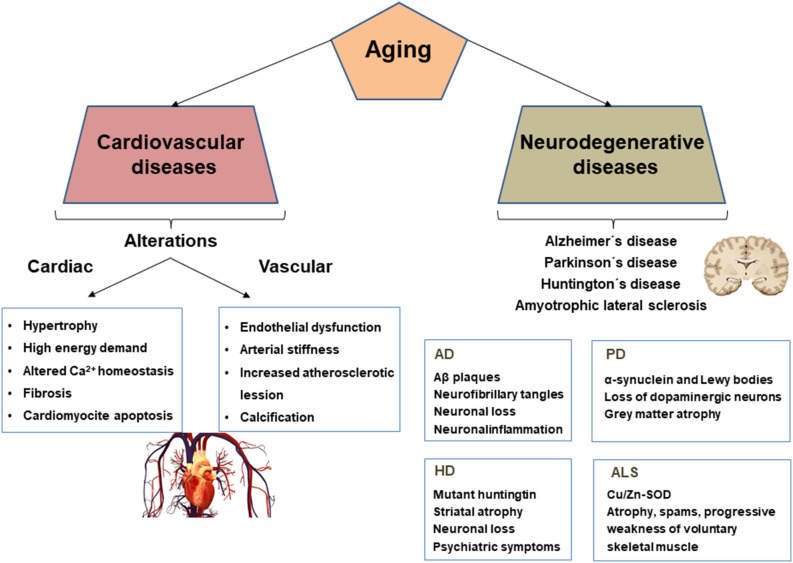
Cardiovascular and neurodegenerative alterations and biomarkers associated with aging. AD, Alzheimer’s disease; PD, Parkinson’s disease; HD, Huntington’s disease; ALS, Amyotrophic lateral sclerosis.

Aging-associated cardiac remodeling is characterized by increased oxygen and energy demand due to elevated afterload and hypertrophy of cardiomyocytes (Lakatta and Levy, 2003b). In addition, fibrosis and abnormal Ca^2+^ handling impair myocardial relaxation and consequently decreases coronary artery perfusion during diastole, thus reducing oxygen supply to the myocardium (Figure 1).

#### Hypertrophy

Cardiac hypertrophy is the response to mechanical stress resulting from an increased workload and is associated with elevated functional demand. Multiple agents are involved in cardiac hypertrophy. Adrenergic activation, catecholamines, angiontensin II (AngII), endothelin 1 (ET-1), and insulin-like growth factor-1 (IGF-1) are well-known neurohumoral factors related to the development of cardiac hypertrophy (Heineke and Molkentin, 2006). Mechanical stress is related to a variety of mechanisms, which include stretch-sensitive ion channels, integrins, and cytoskeletal proteins (Sheydina et al., 2011).

Cardiomyocyte apoptosis is another key feature of age-related heart deterioration. In fact, up to a third of cardiomyocites can be lost as age advances (Centurione et al., 2002). In a mouse model of accelerated senescence (SAMR8), cardiac levels of BAD and BAX increased while Bcl2 levels decreased. In contrast, old SAMR1 animals (senescence acceleration resistant mice) did not show these modifications or showed them at a lower level (Forman et al., 2010). Apoptosis is initiated by intrinsic and extrinsic pathways. The intrinsic route mainly relates to the dysfunction of mitochondria and the endoplasmic reticulum. Apoptosis-inducing factor, endonuclease G, the second mitochondrial-derived activator of caspase (Smac), and cytochrome c are involved in the intrinsic pathway (Sheydina et al., 2011). The extrinsic pathway is activated by factors such as Fas ligand, tumor necrosis factor alpha (TNF-α), TNF-related apoptosis-inducing ligand, as well as caspases 8 and 10 (Desagher and Martinou, 2000). Thus, hypertrophy of the remaining cardiomyocytes as an adaptive mechanism in the aging heart appears to be related to cardiomyocytes loss.

Enlarged cardiomyocytes have an elevated energy demand, and thus they are markedly affected by mitochondrial alterations. Mitochondrial content of cardiomyocytes, which is higher in these cells than in most other cells, decreases with age as does the efficiency of the respiratory chain (Lesnefsky et al., 2016). The aged heart has a reduced capacity for fatty acid use and thus switches to greater utilization of glucose as an energy source (Chia et al., 2018). A similar metabolic shift is also present during heart failure and is accompanied by changes in the expression of metabolic genes (Doenst et al., 2013). Thus, mitochondrial dysfunction in cardiomyocytes represents a pivotal alteration which promotes cardiac aging (Lesnefsky et al., 2016; Gude et al., 2018).

#### Growth

Altered regulation of growth signaling pathways is seen in cardiac hypertrophy and aging. As previously mentioned mTOR integrates nutrient and hormonal signals to regulate growth and has been described as a major modulator of age-related diseases. Animal models documented that increased mTOR signaling impairs resistance to cardiac aging, and suppressing mTOR signaling improves it (Luong et al., 2006).

Insulin and IGF-1 signaling pathways are related to lifespan regulation. The reduction of IGF-1 plasma concentration correlates with an increased risk of heart failure in elderly patients without a previous history of heart disease (Vasan et al., 2003). Additionally, enhanced IGF-1 signaling, such as that occurs as a result of growth hormone (GH) therapy, may be beneficial in heart failure (Khan et al., 2002). The beneficial effects of IGF-1 on cardiovascular disease seem to be due to mitochondrial protection mechanisms. Treatment of cardiomyocytes with IGF-1 decreased mitochondrial superoxide production (Csiszar et al., 2008). Furthermore, low plasma levels of GH and IGF-1 in mice were associated with increased mitochondrial oxidative stress in the vasculature and the heart (Ren and Brown-Borg, 2002).

#### Extracellular Matrix

In the heart and other organs, extracellular matrix (ECM) proteins provide structural support to the adjacent cells. Cardiac ECM proteins including collagen, elastin, fibronectin, laminin, and fibrinogen are produced by fibroblasts. Excessive ECM increases myocardial stiffness, which is a major process responsible for diastolic dysfunction and elevated end diastolic pressure. Age-related cardiac fibrosis seems to be due to dysregulation of ECM synthesis and degradation. ECM production and composition are dynamically controlled by profibrotic factors, matrix metalloproteinases (MMPs), and others. Both connective tissue growth factor (CTGF) and transforming growth factor beta (TGF-β) induce the expression of ECM proteins and inhibit matrix degradation by MMPs (Bujak and Frangogiannis, 2007). In the senescence-accelerated mouse heart, high CTGF and TGF-β expression, cause an imbalance between synthesis and degradation of ECM proteins and is responsible for the enhanced myocardial fibrosis (Reed et al., 2011). MMPs and tissue inhibitors of metalloproteinases (TIMPS) are also altered in the aging heart. MMP-9 levels are increased in the left ventricle and plasma of aged mice (Chiao et al., 2011). In contrast, old MMP-9-knockout mice showed attenuated cardiac aging phenotypes, including reduced collagen deposition and preserved diastolic function. Moreover, reduced expression of profibrotic proteins and CTGF were also observed in those mice (Chiao et al., 2012). Therefore, an imbalance of factors promoting ECM synthesis and degradation underlies the development of cardiac fibrosis in aging hearts.

#### Calcium Homeostasis

Impaired relaxation of cardiomyocytes is another important cardiac alteration in aged hearts and appears to be the main cause of diastolic dysfunction (Borlaug and Kass, 2006). During relaxation, calcium ions dissociate from the actin–myosin complex and are taken up into the sarcoplasmic reticulum and extruded outside the cardiomyocyte by various transporters. Alterations of the calcium cycle and impairment of actin-myosin complex properties lead to impaired cardiomyocyte relaxation (Borlaug and Kass, 2006). Reduced sarcoplasmic reticulum Ca2b-ATPase (SERCA2b) expression and activity (Janczewski and Lakatta, 2010), with a compensatory increase in the levels of the Na^+^/Ca^2+^ exchanger, have been reported in hearts of old mice. Age-related oxidation and nitration of SERCA2b have also been reported (Knyushko et al., 2005). In addition, the aged heart maintains intracellular calcium levels and contractions through a compensatory increase in the activity of L-type calcium channels, and prolongation of action potentials (Josephson et al., 2002; Janczewski and Lakatta, 2010).

#### Neurohormonal Signaling

Dysregulation of both the adrenergic system and the renin-angiotens in-aldosterone axis are considered key regulatory alterations participating in several cardiac disorders. Numerous studies demonstrated that both Ang II and aldosterone induce cardiomyocyte hypertrophy, cardiac fibrosis, and functional alterations (Domenighetti et al., 2005). Inhibition of Ang II metabolism due to the use of an ACE inhibitor or Ang II type I receptor blockers, as well mineralocorticoid receptor antagonism, delays age-dependent cardiac pathological alterations and extend lifespan in rodents (Basso et al., 2007).

Chronic activation of β-adrenergic signaling is deleterious to the heart. Stimulation of the β-adrenergic signaling increases heart rate, contractility, cardiac wall stress, metabolic demand on the heart, and blood pressure (Chiao and Rabinovitch, 2015). These modifications clearly contribute to the development of aged-related cardiac alterations. In fact, deletion of adenylate cyclase type 5, a key enzyme downstream from β-adrenergic signaling, has been shown to extend murine lifespan and is protective against age-dependent cardiac hypertrophy, apoptosis, fibrosis, diastolic, and systolic dysfunction (Yan et al., 2007).

### Aging-Related Arterial Alterations

Aged arteries are characterized by endothelial dysfunction, smooth muscle cell (SMC) migration and proliferation, and vessel wall calcification, leading to progressive arterial stiffness and accelerated atherosclerosis (Figure 1).

#### Endothelial Dysfunction

The vascular endothelium is markedly influenced by aging. The endothelium plays a key role in maintaining vascular homeostasis since it is involved in the control of vascular permeability, regulation of SMC growth, relaxation and contraction, white cell adhesion, platelet aggregation and activation, and blood coagulation, and fibrinolysis. Vascular endothelial cells, through the release of many molecules acting in autocrine and paracrine manners, maintain the function and health of the vascular system (Seals et al., 2011). Reduced availability of NO, either due to diminished production and/or increased NO degradation appears to be the key element in endothelial dysfunction. Reduced endothelial nitric oxide synthase (eNOS) is the most likely mechanism responsible for reduced NO production (Seals et al., 2011). Alterations in eNOS regulatory proteins such as caveolin-1, pAkt, and heat shock protein 90 contribute to the decreased activity of eNOS in aged endothelial cells. The depressed expression of eNOS may also be due to enhanced L-arginine degradation by arginase II, which is overexpressed in advanced age (Donato et al., 2015). As already mentioned, degradation of NO by elevated ROS production is a key mechanism of endothelial dysfunction during aging. Some evidence also indicates that prostanoid vasodilators such as prostacyclin diminish with age. Moreover, reduction of both prostacyclin and NO lead to enhanced ET-1 vasoconstriction, which mediates increased peripheral resistance and hypertension (Donato et al., 2015).

The Framingham Heart Study demonstrated that aging represents the most important independent correlate of endothelial dysfunction (Mitchell et al., 2004). In fact, both elderly men and women showed endothelial dysfunction in the absence of clinical disease. Oxidative stress and inflammation are the most important pathological mechanisms responsible for endothelial dysfunction in older healthy adults (Iantorno et al., 2014). In addition, elevated blood pressure, dyslipidemia, and deglycation exacerbate endothelial dysfunction *via* both oxidative stress and inflammation in advanced aging (Mitchell et al., 2004). Inflammatory factors such as interleukin 6 (IL-6), TNF-α, and monocyte chemoattractant protein-1 (MCP-1), underly chronic the low-grade systemic proinflammatory state correlated with age-associated diseases such as hypertension, obesity, and diabetes (Franceschi and Campisi, 2014). In a model of accelerated senescence, the expression of hemeoxygenases 1 and 2 (HO-1 and 2), proinflammatory cytokines such as TNF-α, IL-1, as well as the expression of iNOS and NF-κB increased significantly, while the expression of eNOS and IL-10 decreased (Forman et al., 2010). This proinflammatory condition together with enhanced ROS production further exaggerates endothelial dysfunction; thus, it is a key feature of endothelial cell senescence.

#### Structure of the Arterial Wall

The normal structure of the arterial wall undergoes structural and functional changes with age, where the wall of the aorta and the central elastic arteries are thickened by the growth of the intima and media layers. This is due to a proliferation of SMC, invasion of the bone marrow by hematopoietic stem cells, an increase in the intercellular matrix and collagenous tissue, as well as fracture of the elastic fibers (Lakatta and Levy, 2003a, b). The imbalance of the elastin/collagen ratio increases arterial stiffness, reduces elasticity and distensibility of the arterial wall, all of which contribute to increased systolic and decreased diastolic blood pressures. Increased media thickness is believed to be a preatherosclerotic state (Bennett et al., 2016). As previously mentioned, endothelial dysfunction is also characterized by elevated intimal permeability, with the accumulation of lipids in the subendothelial space. Under this situation, low-level atherosclerotic stimuli such as mild hypercholesterolemia and/or mild hypertension can easily promote the development of atherosclerotic lesions.

Aged SMC shows a higher proliferative rate than young cells; this correlates with enhanced expression of growth factors. Moreover, an age-related reduction of the anti-proliferative mechanisms of SMC has been also reported (McCaffrey and Falcone, 1993). In addition to structural changes in the arterial media, several paracrine and autocrine factors determine a change in the SMC phenotype, including both contractile to synthetic processes (Owens et al., 2004). These also contribute to increasing proinflammatory factors that promote the progression and development of atherosclerotic lesions. In fact, SMC from old rats presents enhanced expression of inducible NOS (iNOS), monocyte adhesion molecules, and other inflammatory agents (Lakatta, 2015).

#### Atherosclerotic Plaque Morphology

Many reports demonstrated that atherosclerotic lesions from arteries of old individuals are larger than those from younger subjects. Atherosclerotic lesions are more numerous and more vulnerable to rupture in the arteries of older individuals. Reduced fibrous plaque thickness and an increase in the number of inflammatory cells have been observed in atheromatous lesions from aged individuals. These changes were accompanied by an age-associated decrease in SMC content, larger lipid cores, and augmented calcification (Bennett et al., 2016). The increased plaque vulnerability in elderly patients is dependent on mechanisms such as endothelial adhesion of inflammatory cells, increased subendothelial accumulation of atherogenic lipoprotein, increased necrotic lipid core, and calcification of the plaque (Lakatta, 2015).

Atherosclerotic calcification typically increases with age, which suggests a role of calcification in plaque instability (Alexopoulos and Raggi, 2009). The calcification process in aged atherosclerotic lesions appears to be associated with endothelial damage, increased lipoprotein oxidation, and the presence of inflammatory factors (Virmani et al., 2014). There is an important relationship between mortality and the total artery calcium score, and thus it is associated with an increased risk of adverse cardiovascular outcomes (Alexopoulos and Raggi, 2009; Tota-Maharaj et al., 2012). The presence of large amounts of calcium contributes to the rupture of a vulnerable lesion due to the induced changes in mechanical properties of the arterial wall and shear stress effects (Mauriello et al., 2013).

## Neurodegenerative Diseases Related to Aging

Aging is an important risk factor for neurodegenerative diseases and their cognitive deficits. Neurodegeneration is a complicated brain disorder involving so-called biomarkers of aging, along with human genetic and environmental risk factors that are determinants in the onset and progression of neurodegenerative diseases (Wyss-Coray, 2016). Brain aging is an irreversible process, and hallmarks of aging, such as cellular senescence, genomic instability, telomere attrition, mitochondrial dysfunction, and protein aggregation, among others, have been associated with various neurodegenerative diseases, including Alzheimer’s disease (AD), Parkinson’s disease (PD), amyotrophic lateral sclerosis (ALS), and Huntington’s disease (HD; Hou et al., 2019; Figure 1). Brain aging is caused by various morphological, biochemical, metabolic, and circulatory changes, which lead to functional changes, the impact of which depends on the presence or absence of cognitive impairment.

Morphological changes in the brain of elderly people can be observed fundamentally at a microscopic level (number of neurons, dendritic and synaptic loss, and accumulation of disease-specific misfolded proteins in the central nervous system) and are readily apparent in the brain of individuals suffering from neurodegeneration (van Ham et al., 2009; Elobeid et al., 2016). These cellular alterations include intracellular and extracellular deposits of molecular debris and vascular alterations. Thus, Lewy bodies and α-synuclein in PD, β-amyloid peptides, and phosphorylated tau/tau proteins are common in AD (Duong and Gallagher, 1994; Elobeid et al., 2016), superoxide dismutase in ALS (Durham et al., 1997) and mutant huntingtin in HD (Scherzinger et al., 1997). Common pathways involving genes associated with protein degradation have been found in these diseases. Indeed, components of the ubiquitin proteasome system often co-localize with the described pathological protein accumulations in areas of the brain of these patients (Rubinsztein, 2006). Common pathways involving genes associated with protein degradation are frequent in neurodegenerative disorders. Indeed, components of the ubiquitin proteasome system mtypically co-localize with the described pathological protein accumulations in areas of the brain of these patients (Rubinsztein, 2006). Because of this, E3 ubiquitin ligases could target misfolded disease proteins for proteolytic degradation.

The hippocampus, a brain region involved in spatial and episodic memory, may contribute significantly to the decline in cognitive abilities associated with aging (Rosenzweig and Barnes, 2003). Although there is no massive neuronal loss with aging in most brain areas (Rasmussen et al., 1996; von Bohlen und Halbach and Unsicker, 2002), a significant reduction in the number of neurons in the hilum of the hippocampal dentate gyrus has been reported in 24-month-old male rats (Shetty and Turner, 1999). This fact has also been demonstrated from an unbiased stereological analysis to estimate the total number of neurons in the hilum, in male and female Wistar rats of different ages, to confirm the existence of neuronal loss with age (Azcoitia et al., 2005). Neuronal counts were not significantly different between 3 and 22-month-old rats. However, between 22 and 24 months of age, there was a clear deterioration of the structure of the hilum. In addition, this fact was accompanied by an increase in gliosis, which is in agreement with the previous observation of hyperactivation of astroglia and microglia in the hilum of 24-month-old rats (Morgan et al., 1999). We have also detected that there is an important sexual dimorphism in the neuronal content in 24-week-old rats, being greater in males than in females (Azcoitia et al., 2005). Sex differences in hippocampal structure and function may be a consequence of the perinatal effects of testosterone in male rats (Del Cerro et al., 1995).

Neurovascular alterations associated with arteriosclerotic lesions induce hemorrhage and infarction because of the obstruction or rupture of blood vessels, as occurs in multi-infarct dementia or vascular neurocognitive disorder. The biochemical changes in the brain associated with aging are commonly associated with alterations in the levels of various neurotransmitters. Perturbations in the dopaminergic pathway are characteristic of PD, while in AD, the acetylcholinergic pathway in the Meynert’s nucleus is often affected (Nussbaum and Ellis, 2003). Dysregulation of intraneuronal calcium homeostasis, a second key messenger in many neuronal functions, is a common factor in several neurodegenerative disorders. In both AD and PD, alterations have been identified in selective calcium channels and in the intracellular signaling cascade that leads to cell death. In a mouse model for PD, it was possible to prevent the death of dopamine-producing nerve cells by shutting down Cav2.3 channel activity, which has now not been associated with PD (Secondo et al., 2018). In addition, a decrease in the expression of adhesion molecules associated with age has been observed, especially in neurodegenerative diseases; the increased expression of adhesion molecules may be responsible, in part, for the loss of neuronal plasticity in the aged (Fercakova, 2001). Finally, various neurotrophic factors such as the nerve growth factor (NGF), the brain-derived neurotrophic factor (BDNF), and the glial cell line-derived neurotrophic factor (GDNF) are the objects of study in these pathologies (Budni et al., 2015).

In healthy individuals, cerebral blood flow and oxygen consumption remain unchanged during aging. However, in aging, it is very common to find cases of incipient atherosclerosis, which leads to a reduction in blood flow and cerebral oxygen consumption (Bakker et al., 2004). One of the consequences of atherosclerosis involves the possible appearance of small infarcts that translate into ischemic processes that, together with hypoxia and hypoglycemia, produce the activation of neuronal glutamate receptors and the consequent excitotoxicity (Jansen et al., 2014). Likewise, alterations in the blood-brain barrier associated with aging have been observed (Blau et al., 2012); these changes contribute to the development of AD (Takeda et al., 2014). Also, it is documented that in neurodegenerative diseases regional hypometabolism, oxidative stress, and altered metabolism of fatty acids and glucose occur. Therefore, one of the consequences would be a limitation on the ability to adapt to the metabolic stress of glucose deprivation and ischemia, accelerating the development of associated neurodegenerative disease (Yin et al., 2016).

Neurons have high metabolic activity and they require more energy than many other cells to maintain their function, as a result, they are highly sensitive to changes in mitochondrial ATP production. As we previously mentioned, ROS production is significantly increased in damaged mitochondria while energy generation decreases; these changes occur during the aging process itself and in most neurodegenerative diseases (Johri and Beal, 2012). In addition, other functions of the mitochondria in which different intracellular pathways participate are key processes in the development of neurodegenerative diseases (Keogh and Chinnery, 2015). The PINK1/Parkin signaling pathway plays an important role in neuronal mitochondrial dynamics and function, and mutations in either gene cause damage to dopaminergic neurons (Shiba-Fukushima et al., 2017). Moreover, mitophagy is a form of autophagy that selectively degrades dysfunctional mitochondria and has a key role in preventing age-related diseases. This process is important for mitochondrial quality control, and to reduce or dampen inflammation, a process that is common in neurodegeneration. Extensive evidence suggests that defects in the process of selective autophagy of dysfunctional mitochondria contribute to neurodegeneration (Fivenson et al., 2017), as well as alterations in the mitochondrial unfolded protein response (UPR), which is a signal transduction pathway from mitochondria to the nucleus.

It also is important to take into account the epigenetic mechanisms that contribute to the development of neurodegenerative diseases, such as DNA methylation, changes in chromatin structure, mainly due to histone acetylation, ubiquitination, and phosphorylation mechanisms, as well as non-coding ribonucleic acids (micro-RNAs; López-Otín et al., 2013). Telomere shortening or instability may also participate in the development of these neurodegenerative disorders. In studies carried out in mice and humans, it was observed that the shortening of telomeres accelerated aging, and in patients with AD, these alterations are associated with a cognitive deficit, β-amyloid aggregates, and phosphorylated tau associated with oxidative and inflammatory processes (Herrmann et al., 2018).

Alzheimer’s disease is the leading cause of dementia in the world, accounting for 80% of cases and one of the great health challenges in the 21st century. The main risk factor is age, usually affecting people over 65 years of age (Scheltens et al., 2016). Alzheimer-type dementia is a neurodegenerative process that affects the brain areas involved in the memory and behavior processes, therefore, this pathology is characterized by the progressive loss of memory and cognitive abilities, beginning with retrograde amnesia and can ultimately lead to death (Citron, 2002). The pathophysiological effects of AD are characterized by a progressive and bilateral irreversible atrophy of brain volume due to the appearance of selective neuronal degeneration, mainly in areas of the brain associated with cognition and memory, with low levels of some neurotransmitters, such as acetylcholine (Nussbaum and Ellis, 2003). In AD patients, along with senile plaques (β-amyloid peptides) and neurofibrillary tangles (tau/phosphorylated tau proteins), inflammatory processes, oxidative stress, mitochondrial dysfunction, and the appearance of reactive gliosis (Ransohoff, 2016) are also evident. Defect in amyloid precursor protein (APP), presenilin 1 (PSEN1), presenilin 2 (PSEN2), and apolipoprotein E4 (APOE4) are central risk factors for AD (Bekris et al., 2010). The concentration of β-amyloid peptide is regulated by degrading enzymes including neprilysin, insulin-degrading enzyme, Ang I converting enzyme (ECAI), and by the process of endocytosis mediated by microglial cells present in the blood-brain barrier, one of the most important in the elimination of this peptide. Therefore, even a small reduction in the rate of degradation or clearance of β-amyloid peptide, as well as an increase in its production, leads to its pathological accumulation. Likewise, tau hyperphosphorylation reduces its affinity for neuronal microtubules, causing the loss of their functionality. This leads to a pathological alteration of the structural and regulatory functions of the neuronal cytoskeleton, favoring the development of neurodegenerative processes and the formation of neurofibrillary tangles (Figure 1).

Parkinson’s disease is the second most common neurodegenerative disease associated with aging. Clinical signs of this pathology include resting tremor, rigidity, and bradykinesia, which are made evident by the loss of about 80% of striatal dopamine and 50% of nigral neurons (Fearnley and Lees, 1991). As in AD, age is the greatest risk factor for the onset and progression of PD (Hindle, 2010). In patients in whom this pathology began after the age of 70, dementia occurred earlier and the accumulation of α-synuclein and Lewy bodies was greater than in younger-onset PD patients (Halliday and McCann, 2010). This disease is characterized by the loss of dopaminergic neurons in the substantia nigra pars compacta that project to the striatum (Samii et al., 2004), and the early accumulation of α-synuclein deposits in the cholinergic neurons of the basal forebrain. This protein is misfolded and accumulates to form Lewy bodies (van Ham et al., 2009), and other mechanisms such as oxidative stress, mitochondrial dysfunction, disrupted axonal transport, glutamate neurotoxicity, cellular senescence, and neuroinflammation are included in its pathogenesis (Poewe et al., 2017). Autopsy examinations on tissue samples from PD patients have shown glial cell activation, α-synuclein aggregates, reduced SIRT1 activity, and increased expression of proinflammatory cytokines, such as IL-1β, which requires activation of the NLRP3 inflammasome for maturation and activation (Codolo et al., 2013). Toll-like receptors (TLR) and NF-kB participate in this activation process, and α-synuclein aggregates exert deleterious effects on synaptic function and mitochondrial homeostasis, in addition to inducing microglial activation excessive (Figure 1). All this would contribute to neuroinflammation and neuronal death.

While Alzheimer’s and Parkinson’s diseases are the most prevalent neurodegenerative pathologies in the aged, there are others that have a strong link with aging, including ataxia telangiectasia, ALS, and HD. Ataxia telangiectasia is a rare hereditary disease that manifests in childhood. It is a rare disorder characterized by the association of severe combined immunodeficiency, mainly affecting the humoral immune response, with progressive cerebellar ataxia. It includes neurological signs, telangiectasia, increased susceptibility to infection, and an elevated risk of developing cancer (Shiloh and Kastan, 2001). ALS is a neurodegenerative disorder characterized by the loss or dysfunction of motor neurons in the central nervous system, and multiple genetic mutations in non-neuronal cells have been identified (Rowland and Shneider, 2001). ALS patients suffer from atrophy, spasms, and progressive weakness of voluntary skeletal muscles. One of the possible mechanisms responsible for the pathophysiology could be associated with a deficiency in mitochondrial transport or mitophagy since it could increase the accumulation of dysfunctional mitochondria in the axon of motor neurons (Menzies et al., 2002). This would alter the activity of the electron transport chain and affect the production of ATP. Furthermore, mitochondrial analysis in ALS patients showed impaired calcium homeostasis, leading to increased oxidative damage associated with ROS production in motor neurons (Menzies et al., 2002; Figure 1). Moreover, PPARγ could be part of the main signaling pathway involved in neuroinflammation in ALS (Skerrett et al., 2014). Finally, HD is an inherited neurodegenerative disease, autosomal dominant and age-dependent, which is caused by a mutation in the huntingtin protein. This disease is characterized by aberrant motor, cognitive and behavioral symptoms. The mutated huntingtin protein accumulates and forms cytoplasmic and nuclear inclusions, alters gene transcription, decreases mitochondrial activity, and causes activation of proapoptotic molecules (Scherzinger et al., 1997; Figure 1). There is also an increase in excitotoxicity, altered proteasomal function, production of oxidative damage, and metabolic alterations, due to the accumulation of mutated huntingtin inside cells (Cattaneo et al., 2001).

## Melatonin and Its Relationship with Aging

**Figure 2:**
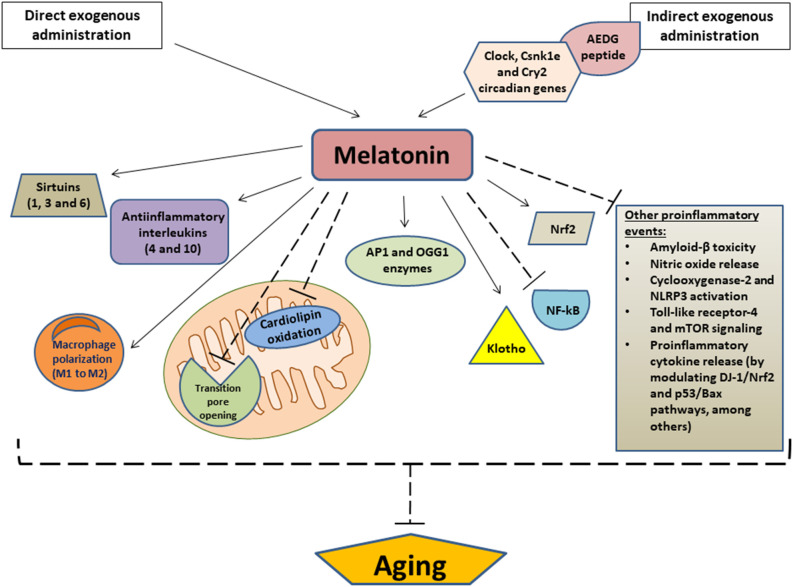
Relevant anti-aging effects of melatonin. Arrows indicate stimulation and dashed lines indicate inhibition. AEDG, (Ala-Glu-Asp-Gly) peptide; APE1, Apurinic/apyrimidinic endonuclease 1; OGG1, (8-Oxoguanine glycosylase); Nrf2, Nuclear factor erythroid 2-related factor 2; NF-κB, nuclear transcription factor kappa B; NLRP3, NOD-like receptor family pyrin domain-containing 3; mTOR, mammalian target of rapamycin.

As noted, aging is closely related to mitochondrial dysfunction while long-term treatment of aged rats with melatonin enhanced mitochondrial function by both preventing the opening of permeability transition pore in the inner mitochondrial membrane and retaining the cytochrome c and 2’,3’-cyclic nucleotide 3’-phosphodiesterase inside mitochondria, thus providing protein protection against harmful effects of 2’,3’-cAMP (Baburina et al., 2017). Melatonin also prevents mitochondrial dysfunction and cellular aging by limiting the oxidation of cardiolipin, a phospholipid localized in the inner mitochondrial membrane. Cardiolipin plays a key role both in multiple mitochondrial bioenergetic mechanisms and in the stability and dynamics of the mitochondrial membrane, as well as in several apoptotic events that involve the mitochondria (Paradies et al., 2017; Figure 2).

Other mechanisms by melatonin which exerts anti-aging effects are the modulation of the sirtuin1 (a deacetylase that regulates metabolic activity in response to cellular stress) pathway and the modulation of autophagy, which is decreased during aging. Autophagy is a natural cellular homeostatic mechanism of removing damaged cells, in order to regenerate newer, healthier cells. Therefore, to enhance autophagic processes is crucial to prevent or attenuate cellular aging. In this regard, it was observed that melatonin may increase autophagic activity *via* sirtuin1 in different aging models, which may be therapeutically useful for the prevention and treatment of multiple age-related diseases (Nopparat et al., 2017; Boga et al., 2019). Melatonin also collaborates with sirtuin 3, another NAD^+^-dependent deacetylase which, like sirtuin 1, regulates the mitochondrial redox state, among many other functions. Together, melatonin and sirtuin 3 influences mitochondria dynamics and scavenges free radicals, thus preventing or delaying cellular aging and its derived diseases, which are usually developed as a consequence of a redox imbalance (Reiter et al., 2018). In fact, it was observed that the expression patterns of melatonin and different sirtuins (sirtuin 1,3, and 6) significantly decreased in samples of buccal epithelium from old patients compared to young patients, and even more evidently in those who suffer from hypertension. This finding reinforces the likely relationship between age-related diseases (such as hypertension) and melatonin levels (Carbone et al., 2020; Figure 2).

In addition to its action on sirtuins, melatonin may also upregulate Nrf2 (Nuclear factor erythroid 2-related factor 2), an antiinflammatory transcription factor) and downregulate NF-κB (a proinflammatory transcription factor) to attenuate inflammatory processes at the cellular level. Likewise, this indoleamine also stimulates the release of anti-inflammatory cytokines such as IL-4 and IL-10, as well as promoting the polarization of macrophages from a proinflammatory phenotype (M1 phenotype) to an anti-inflammatory phenotype (M2 phenotype). Additionally, melatonin suppresses other proinflammatory events, such as β- amyloid toxicity, NO release, cyclooxygenase-2, and NLRP3 inflammasome activation, TLR4, and mTOR signaling, and cytokine release by senescence-associated secretory phenotype, among others. Together, these actions significantly contribute to the anti-aging effect of exogenous melatonin, since aging and its related pathologies are usually associated with chronic proinflammatory processes, which are a consequence, at least in part, of reductions in endogenous melatonin secretion (Hardeland, 2019). Similarly, a study revealed that the exogenous administration of this compound was able to reduce serum levels of proinflammatory cytokines, activate the DJ-1/NRFrf2 antioxidant signaling pathway, and inhibit p53/Bax apoptotic pathway in an animal model, thus counteracting the aging-derived changes related to these pathways (Ismail et al., 2018). It has also been reported that melatonin upregulates the expression of Klotho, an important anti-aging protein with powerful antioxidant, anti-inflammatory, and antiapoptotic properties (Ko et al., 2019). Of special interest, another study demonstrated that exogenous melatonin is effective in decreasing DNA damage caused by aging, presenting antigenotoxic and antimutagenic effects in old Swiss mice. The animals treated with melatonin for 18 months showed high levels of APE1 (Apurinic/apyrimidinic endonuclease 1) and OGG1 (8-Oxoguanine glycosylase), two important enzymes involved in the base excision repair pathways of DNA lesions. Therefore, melatonin showed to have efficient pharmacological properties which aid in the modulation of genetic and physiological alterations derived from aging (Damiani et al., 2020). Another important finding to highlight is that melatonin supplementation maintains the normal redox homeostasis in old animals exposed to artificial light. This is of great importance because artificial light exposure at night may induce oxidative stress due to the alteration of the circadian cellular physiology as a consequence of the reduction in circulating levels of melatonin. Therefore, melatonin supplementation would represent a useful strategy to alleviate age-related circadian alterations, particularly in light-polluted areas (Verma et al., 2020; Figure 2).

However, the anti-aging effects of melatonin may not only be observed using exogenous melatonin as a therapeutic agent, but also by administering other compounds that may stimulate or induce endogenous melatonin release. For instance, the AEDG (Ala-Glu-Asp-Gly) peptide, has demonstrated promising geroprotective/anti-aging actions due to its ability to restore the pineal melatonin production by regulating the expression of multiple human circadian genes, such as Clock, Csnk1e and Cry2, among others (Ivko et al., 2020; Figure 2).

## Melatonin as A Therapeutic Agent Useful in The Treatment of Aging-Derived Cardiovascular and Neurodegenerative Pathologies

### Cardiovascular Diseases

An exhaustive analysis carried out on cardiac cells of aged rats with acute heart failure showed that chronic intake of melatonin reduced the alterations associated with age in the muscle cell structure of the left ventricle; this treatment also reduced muscle fiber swelling, and amount of damaged tissue. In addition, melatonin administration improved the respiratory control index, the mitochondrial Ca^2+^ retention ability, and diminished mitochondrial swelling in the cardiac cells of these animals. Finally, melatonin partially prevented the alterations at the subunit level of respiratory complexes III and V and significantly reduced the expression of complex I subunit NDUFB8 in heart mitochondria of the treated animals, thus inhibiting ROS generation. Due to these actions, exogenously-administered melatonin prevented the mitochondrial dysfunction associated with heart failure and retained the levels of 2’,3’-cyclicnucleotide-3’-phosphodiasterase (anti-aging enzyme) in isolated heart mitochondria of treated animals (Odinokova et al., 2018). Melatonin treatment also aided the recovery of mitochondrial dynamics in a mouse model of myocardial aging, providing an anti-apoptotic action, restoring Nrf2-antioxidant capacity, and enhancing mitochondria ultrastructure impaired by aging (Fernández-Ortiz et al., 2020). Because aging also predisposes to the development of lethal cardiac arrhythmias and melatonin is a potential anti-aging agent, this indoleamine may be further considered as an anti-arrhythmic molecule (Segovia-Roldan et al., 2021). Melatonin administration to aged mice also demonstrated the therapeutic utility of this compound in the treatment of age-related cardiac sarcopenia, since it preserved the normal cardiomyocyte structure and restored their number, as well as to reducing β-myosin heavy chain expression (a marker of cardiac hypertrophy) in these animals. Moreover, melatonin stimulated mitochondrial architecture recovery, decreased apoptosis and formation of multivesicular bodies, and reduced the expression of proinflammatory cytokines including IL-1α and IL-6 (Sayed et al., 2021).

Of interest, the activation of the NADPH oxidase pathway by angiotensin II significantly promotes DNA damage and accelerates both aging and the appearance of multiple age-related pathologies, especially cardiovascular disorders. In relation to this, it has been suggested that melatonin could alleviate oxidative stress induced by angiotensin II through the activation of the mitochondrial calcium uptake 1 pathway, which is crucial in the prevention of mitochondrial calcium overload that may trigger exacerbated generation of damaging ROS (Sehirli et al., 2021).

Regarding the effects of exogenous melatonin on aged myocardium subjected to ischemia/reperfusion injury, a study carried out on aged rats showed that this treatment reduced infarct size in comparison with the control group. In addition, melatonin upregulated the Bcl-2, sirtuin 3, and FOXO type 1 gene expression, as well as downregulated the Bax and caspase-3 gene expression. Therefore, melatonin may protect the aged heart against ischemia/reperfusion injury by inhibiting apoptosis and activating the sirtuin 3/FOXO1 signaling pathway (Jafari-Azad et al., 2021). A similar study also showed cardio protective effects of melatonin against ischemia/reperfusion injury in aged hearts because it lowered oxidative stress, enhanced mitochondrial membrane potential, and restored the NAD^+^/NADH ratio (Hosseini et al., 2020).

Exogenous melatonin also caused a significant reduction in oxidative stress and inflammation in the vasculature of a mouse model of age-related vascular dysfunction. Likewise, this treatment restored the normally weak contractile actions of the perivascular tissue usually implicated in the development of vascular dysfunction, a condition observed in multiple cardiovascular pathologies associated with aging (Agabiti-Rosei et al., 2017). As an additional finding, one report observed that both osteogenic differentiation and senescence of cultured vascular SMC were reduced by melatonin treatment through the activation of melatonin receptors, and involved a paracrine mechanism dependent on exosomes secreted by these cells. This study also showed that the paracrine effect was mediated by exosomal miR-204/miR-211. Therefore, microRNA exosomes from vascular SMC treated with melatonin reduced both vascular calcification and vascular aging, thus preventing or delaying the development of multiple cardiovascular diseases related to both processes (Xu et al., 2020).

Atherosclerosis is a common cardiovascular disease associated with aging, which is related, at least in part, to telomerase dysfunction. In this regard, it was proven that melatonin treatment prevented all the altered parameters related to aging at the cardiovascular level in an atherosclerotic aged-mouse model, such as increased ROS, malondialdehyde, and inflammatory cytokine levels, as well as cell apoptosis. Melatonin’s ability to beneficially upregulate the activity of telomerase, explains its actions in preventing or attenuating cardiovascular dysfunction and exerting important anti-aging effects on the blood vessels (Xie et al., 2021). It has been widely documented that melatonin exerts its protective effect as a result of its antioxidant scavenging properties, its direct free radical detoxification, and its ability to preserve efficient oxygen metabolism in the mitochondria (Martinez-Cruz et al., 2002). Melatonin has also been shown to be effective at the cardiovascular level in lowering blood pressure in humans, type 1 diabetic adolescents (Cavallo et al., 2004), and postmenopausal women on hormone replacement therapy (Cagnacci et al., 2001).

Another study showed that melatonin is a valuable therapeutic agent against ischemic stroke related to aging, demonstrating that aged Wistar rats treated with melatonin from 24 h prior to until 7 days after an induced ischemic stroke had a significant reduction in the concentrations of glial fibrillary acidic protein, TNF-α, IL-1β, Bcl-2-associated death promoter, and Bcl-2-associated X protein in both the hippocampus and brain cortex, while the hippocampal concentrations of sirtuin 1 and Bcl-2 were increased. Melatonin treatment only following the induction of ischemic stroke resulted in similar protective effects, and were much less significant compared to the group that also received melatonin pre-treatment (Rancan et al., 2018). In the previously described study with accelerated senescence mice (SAMP8), administration of melatonin for 30 days was able to counteract changes in the cardiac expression of inflammatory mediators (TNF-α, IL-1β, and IL-10, NFkBp50 and NFkBp52), markers of apoptosis (BAD, BAX, and Bcl2) and parameters related to oxidative stress (HO-1 and 2, iNOS and eNOS; Forman et al., 2010). Although in some cases the highest dose of the hormone (10 mg/kg/day) showed a more marked effect (e.g., on eNOS, HO-1, and Bcl2) as compared with the lower dose (1 mg/kg/day), the lower dose has shown also, a profound protective effect on cardiac aging in these animals (Forman et al., 2010). These results suggest that chronic administration of melatonin was able to reduce the increase in inflammation, apoptosis, and oxidative stress related to age, especially in the group of animals with a higher degree of cardiovascular alterations (Figure 2).

### Neurodegenerative Pathologies

Melatonin may act as a protective molecule against aging-derived neurodegenerative diseases by preserving the integrity and permeability of blood-brain barrier through different mechanisms. These include the inhibition of NADPH oxidase-2 and matrix metalloproteinase-9, the regulation of sirtuin 1 and NLRP3 inflammasome, the activation of AMP-activated protein kinase, and the inhibition of gp91phox, among others (Liu et al., 2017). Melatonin also was found to be useful in the protection against age-dependent neuronal oxidative stress induced by artificial light at night in the brain of chrono-disrupted rats. These beneficial actions of melatonin involved anti-oxidative, anti-inflammatory, and mitochondrial protective effects, which prevented neuronal degeneration in these animals (Verma et al., 2021).

Regarding the anti-neuroaging mechanisms of melatonin related to sirtuins, aged mice treated with this indoleamine showed, at the hippocampal level, a significant increase in the expression of sirtuin 1, FOXO1, and FOXO3a, which promotes anti-aging actions by regulating the cell cycle. Likewise, melatonin treatment caused to arise in melatonin receptors 1 and 2, which were down-regulated in these animals. Furthermore, the aged mice also experienced a significant reduction in the expression of p53, ac-p53, MDM2 (a negative regulator of the p53 tumor suppressor), and DKK1 (usually induced by the accumulation of amyloid-β), which were increased due to the aging process. Moreover, this study suggested that these anti-neuroaging gene effects of melatonin are possibly mediated by neuropeptide Y (Jenwitheesuk et al., 2017, 2018). For its part, the administration of melatonin to aged Wistar rats caused a decrease in sirtuin 2 and FOXO3a expression (which were found increased in these animals, probably in an attempt to counteract harmful effects of aging), as well as a reduction in oxidative stress parameters and pro-apoptotic proteins. Measurements of nucleosomes in brain homogenates showed an age-related increase that was accompanied by a decrease in Bcl2, a protein that protects against apoptosis. The increase in nucleosomes indicating increased apoptosis was also associated with an increase in caspases 3 and 9. After melatonin treatment, a clear inhibition of apoptosis was observed, which was accompanied by decreased levels of nucleosomes and an increase in Bcl2 levels (Azcoitia et al., 2005). Likewise, melatonin treatment provoked an increase in Bcl2, Bcl2/Bax, and total antioxidant status at the hippocampus level, which were found to be decreased in the aged brain of these animals (Keskin-Aktan et al., 2021). It has also been suggested that another neuroprotective action of melatonin against aging-related neurodegenerative diseases is the restoration of the phosphorylation/dephosphorylation rate, which is commonly altered during some of these pathologies. In this regard, it has been proposed that melatonin probably stimulates the activity of phosphoprotein phosphatases enzymes (in charge of phosphorylation/dephosphorylation processes) through direct interaction with them (Arribas et al., 2018; Figure 2).

Stroke is one of the most important causes of morbidity and mortality in the world, and aging is one of the risk factors that significantly increase its incidence. Due to the inflammatory and apoptotic response to ischemia, together with the increased release of ROS, the affected brain tissue might lead to several pathological alterations. Blockade of the right middle cerebral artery as a model of ischemic brain injury in old rats significantly increased the expression of IL-1β, TNF-α, Bax, and glial fibrillary acidic protein in the hippocampal and cortical areas. These effects were markedly more deleterious and were aggravated in old rats. Melatonin treatment significantly decreased the expression of these markers in the ipsilateral and contralateral ischemic areas of the right and left hippocampus and cortex (Paredes et al., 2015). Likewise, melatonin reduced infarct volume and the apoptotic response resulting from cerebral ischemia and reperfusion, preventing ischemic brain injury through activation of the mTOR/p70S6 kinase signaling pathway (Koh, 2008). Several studies have unequivocally supported the idea that sirtuins have therapeutic potential in neurodegenerative diseases such as stroke or ischemic brain injury (Raghavan and Shah, 2012). SIRT1 has also been linked to reduced apoptosis, while its inactivation appears to promote Bax translocation from the cytosol to mitochondria (Cohen et al., 2004). In old rats, we showed that SIRT1 expression was reduced in the dentate gyrus and that its expression was increased after melatonin treatment. Furthermore, melatonin was able to counteract the hippocampal decrease of SIRT1 due to ligation of the middle cerebral artery (Paredes et al., 2015). These results support previous studies in which melatonin, through the activation of the SIRT1 pathway, prevents and protects against neurodegenerative processes, since the benefits include the total preservation of cognitive capacity, protection against amyloid and tau pathology, activation of abnormal protein pathways, and activation of neurotrophy (Sarlak et al., 2013; Figure 2).

As mentioned above, the pathogenesis of AD, one of the most prevalent aging-related neurodegenerative pathologies worldwide involves multiple neuronal impairments which result in sleep disruption, circadian alterations, and mitophagic (mitochondrial autophagy) dysfunction, among others. In this context, melatonin not only has the ability to ameliorate sleep and circadian disorders but also may successfully remove the misfolded protein aggregations formed during this neurodegenerative pathology through its mitophagy-regulatory actions (Spinedi and Cardinali, 2019; Luo et al., 2020; Hossain et al., 2021).

In relation to memory impairment associated with AD and other neurodegenerative pathologies, melatonin treatment also showed a significant reduction in neuroinflammation in memory-related areas of the brain of aged mice, which may be useful in the prevention of this condition during aging. Moreover, melatonin caused a reduction in protein levels of glial fibrillary acidic protein, integrin αM, the major pro-inflammatory cytokines (IL-1β, IL-6, and TNF-α) and phosphor-NFκB at the brain level. Likewise, melatonin increased brain-derived neurotrophic factor, Ca^2+^/calmodulin-dependent protein kinase II and N-methyl-D-aspartate receptor subunits NR2A and NR2B, which were downregulated in different memory-related areas of the brain in these aged animals (Permpoonputtana et al., 2018). In addition, N1-acetyl-5-methoxykynuramine (a metabolite produced in the brain after exogenous melatonin crosses the blood-brain barrier) also improved long-term object memory performance in aged mice (Iwashita et al., 2021). Therefore, melatonin treatment may prevent or attenuate memory decline, which, as mentioned, is a common feature in multiple neurodegenerative diseases associated with aging. Thus, this indoleamine could promote neuroprotective effects and enhance not only memory but also of learning during aging (Hosseini et al., 2019).

Regarding other neurodegenerative diseases in which therapeutic use of melatonin was tested, in a mouse model of accelerating aging with HD, the aging process increased oxidative stress and reduced mitochondrial membrane potential, which stimulated higher release of mitochondrial DNA at the neuronal level. The mitochondrial DNA discharge into the cytosol activated the cGAS/STING/IRF3 signaling pathway, thus promoting the production of inflammatory cytokines and, consequently, the development of this neurodegenerative disorder; melatonin treatment inhibit these alterations thus avoiding the neuroinflammaging process (Jauhari et al., 2021). For its part, a case-control study found that melatonin levels were altered in aged patients with PD and this reduction may be useful as endogenous markers for sleep and wakefulness disturbances of this prevalent aging-related neurodegenerative disease (Li et al., 2021). Notably, sleep behavior disorder in patients with PD may precede the development of motor symptoms and is a marker of a worse prognosis for this pathology. In this regard, it has been demonstrated that daily bedtime administration of 3–12 mg of melatonin is effective in the treatment of sleep behavior disorders and may stop or slow neurodegeneration associated with these diseases. However, if doses of melatonin that have been effective in animal models to reduce not only sleep disorders but also the symptomatology of PD are projected to humans, these would be in the range of 40–100 mg/day, a dose that is consistent with studies in which melatonin was used to inhibit COVID-19 infections (Reiter et al., 2022). Clearly, clinical studies using this dose range are urgently needed to provide information on the optimal dose of melatonin for human use in relation to the treatment of symptomatology of this and other neurodegenerative diseases (Cardinali, 2019; Pérez-Lloret and Cardinali, 2021).

## Conclusion and Prospects

Mitochondria present a paradox in terms of their physiology. They are a major generator of damaging free radicals and, at the same time, they have the responsibility of supplying ATP and may also be a source of melatonin. Therefore, the development and progression of aging-derived processes and the related diseases depend on the maintenance of an adequate mitochondrial function and, consequently, likely on an appropriate oxidative stress/melatonin level ratio. A number of experimental studies indicate that melatonin has indicated that its chronic administration may slow some aspects of aging, e.g., reducing the oxidative stress load; but the cytoprotective/anti-aging effects of melatonin still require the definition of an optimal daily dose to achieve these benefits. The clinical trials related to the potential therapeutic use of melatonin to alter aging processes in humans have not used especially high doses. The dose issue is generally considered to not be a major problem when melatonin is used because of its uncommonly low toxicity and its safety over a very large dose range.

Melatonin has several physicochemical and pharmacokinetic features that may influence its therapeutic use. Thus, its rapid clearance from the blood has been mentioned as a limiting feature. However, blood levels are far less important than intracellular concentrations so the rapid uptake of melatonin from the blood may not be a limitation. Also, since only an estimated 5% of the total melatonin produced in the body is derived from the pineal gland (Reiter et al., 2020); its inhibition in this organ by light at night may not be a major shortcoming. The bulk of the melatonin produced in mammals is believed to be from the mitochondria of every cell, where its synthesis presumably occurs 24 h a day and is not impacted by the light/dark cycle (Suofu et al., 2017). Importantly, the availability of nano-encapsulated melatonin preparations which are available will likely become highly advantageous. Their regular use could well improve availability and sustain intracellular concentrations at a level where they would provide health advantages against aging-derived pathologies, including cardiovascular and neurodegenerative diseases (Sarkar et al., 2017; Martín Giménez et al., 2020; Chuffa et al., 2021).

## Author Contributions

VM, NH, VL, and WM drafted the manuscript. VM, NH, VL, JT, RR, and WM reviewed, discussed, and performed the critical editing. All authors contributed to the article and approved the submitted version.

## Conflict of Interest

The authors declare that the research was conducted in the absence of any commercial or financial relationships that could be construed as a potential conflict of interest.

## Publisher’s Note

All claims expressed in this article are solely those of the authors and do not necessarily represent those of their affiliated organizations, or those of the publisher, the editors and the reviewers. Any product that may be evaluated in this article, or claim that may be made by its manufacturer, is not guaranteed or endorsed by the publisher.
